# A Novel Reaction of Peroxiredoxin 4 towards Substrates in Oxidative Protein Folding

**DOI:** 10.1371/journal.pone.0105529

**Published:** 2014-08-19

**Authors:** Li Zhu, Kai Yang, Xi’e Wang, Xi Wang, Chih-chen Wang

**Affiliations:** 1 National Laboratory of Biomacromolecules, Institute of Biophysics, Chinese Academy of Sciences, Beijing, China; 2 University of Chinese Academy of Sciences, Beijing, China; Instituto de Biociencias - Universidade de São Paulo, Brazil

## Abstract

Peroxiredoxin 4 (Prx4) is the only endoplasmic reticulum localized peroxiredoxin. It functions not only to eliminate peroxide but also to promote oxidative protein folding via oxidizing protein disulfide isomerase (PDI). In Prx4-mediated oxidative protein folding we discovered a new reaction that the sulfenic acid form of Prx4 can directly react with thiols in folding substrates, resulting in non-native disulfide cross-linking and aggregation. We also found that PDI can inhibit this reaction by exerting its reductase and chaperone activities. This discovery discloses an off-pathway reaction in the Prx4-mediated oxidative protein folding and the quality control role of PDI.

## Introduction

Many cellular activities generate reactive oxygen species (ROS) which can function as signaling molecules, however, overproduction of ROS may result in oxidative damage contributing to diseases and ageing [1]. In the endoplasmic reticulum (ER), disulfide formation in secretary and membrane proteins is primarily catalyzed by the Ero1 oxidase/protein disulfide isomerase (PDI) pathway, which is accompanied by the production of hydrogen peroxide (H_2_O_2_) [2], [3]. How the ER prevents H_2_O_2_ accumulation has become the subject of extensive investigations in recent years.

The peroxiredoxins (Prxs) are a group of cysteine-based peroxidases, which eliminate peroxide and regulate its signaling in the cells [4]. Typical 2-Cys Prxs contain two conserved cysteine residues responsible for peroxidase activity. One is the peroxidatic cysteine (CysP), which reacts with H_2_O_2_ to form sulfenic acid (CysP-SOH), and further reacts with the other one, the resolving cysteine (CysR), to form an intersubunit disulfide bond [5]. Prx4, a mamalian 2-Cys Prx, is the only ER-located Prx reported to date [6], and has been characterized to be an efficient H_2_O_2_ scavenger [7]. It was recently reported that Prx4 oxidized by H_2_O_2_ can transfer its disulfide to PDI [8], a key foldase and chaperone in the ER [9], [10], which further oxidizes folding substrates. The Prx4/PDI system was thus established to be a new oxidative folding pathway in parallel and coupling with the Ero1/PDI pathway. The two pathways together can generate two disulfide bonds and two H_2_O molecules at the expense of a single O_2_ molecule, avoiding the release of peroxide [11], which is the by-product in the Ero1/PDI pathway. The importance of the two pathways was further confirmed in a recent study employing a combined knockout of both Prx4 and Ero1 [12].

However, the detailed mechanism of the Prx4-mediated oxidative folding pathway, i.e. the interactions between various components, remains largely unknown. We thus reconstituted an *in vitro* oxidative protein folding system composed of H_2_O_2_, Prx4, PDI and a denatured and reduced substrate to study the interactions involved. Here we report an unexpected finding that Prx4 can directly react with folding substrates via CysP, which results in disulfide cross-linking and aggregation. This finding discloses an off-pathway in the Prx4-mediated oxidative folding pathway. We also identified a role of PDI in inhibiting the disulfide cross-linking reaction and aggregation, in addition to mediating disulfide transfer from Prx4 to substrates. Both the reductase and the chaperone activities of PDI contribute to the quality control of Prx4-mediated oxidative protein folding.

## Materials and Methods

### Protein expression and preparation

The coding sequences of mature human Prx4, PDI and *Escherichia coli* thioredoxin (Trx) were cloned into pQE-30 (Qiagen), and all the resulting proteins contain N-terminal (MRGSH_6_GS-) tags. Prx4 mutants were constructed by overlap extension PCR, and verified by DNA sequencing.

The proteins were expressed in *E. coli* strain M15 [pREP4] (Qiagen). Cells were grown at 37°C in LB medium containing 100 µg/ml ampicillin, and isopropyl β-D-thiogalactoside was added to a final concentration of 1 mM at A_600_ of ∼0.6. After shaking for additional 4 h, the cells were harvested and lysed for protein purification with a nickel-chelating column (GE Healthcare). The elute was dialyzed against buffer A (50 mM Tris-HCl buffer containing 150 mM NaCl, pH 7.6) and stored as aliquots at −80°C.

Trx at ∼500 µM was reduced by 100 mM dithiothreitol (DTT) at 25°C for 1 h as previously described [2]. Reduced Prx4-C14S was prepared by incubating 100 µM Prx4-C14S with 20 mM DTT at 25°C for 1 h, followed by buffer exchange using a HiTrap desalting column (GE Healthcare) into buffer A. Oxidized Prx4-C14S was produced by incubation of 50 µM Prx4-C14S with 100 µM H_2_O_2_ at 25°C for 5 min. Protein concentrations were determined spectrophotometrically at 280 nm with the absorption coefficient of 36900 M^−1^·cm^−1^ for Prx4 and its mutants and 13980 M^−1^·cm^−1^ for Trx. The concentrations of PDI and its mutants were determined by Bradford assay with bovine serum albumin as a standard.

### Assays in the reconstituted Prx4-mediated oxidative folding system

Denaturation and reduction of RNase A and bovine pancreatic trypsin inhibitor (BPTI) were carried out basically as described [13]. Prx4-mediated protein refolding in the reconstituted system was initiated by adding H_2_O_2_ to a final concentration of 50 µM into buffer B (100 mM Tris-HAc buffer with 50 mM NaCl and 1 mM EDTA, pH 8.0) containing 2.5 µM Prx4, 2.5 µM PDI and 8 µM denatured and reduced RNase A (drRNase A) as a substrate at 25°C. For analysis of the redox states of RNase A during refolding, aliquots of 40 µl were removed at different time points and quenched with 10 µl of 10 mM 4-acetamido-4′-maleimidylstilbene-2,2′-disulfonic acid, followed by non-reducing SDS-15% PAGE and silver staining for good resolution of RNase A bands at different redox states. Reactivation of drRNase A was assayed in the same reconstituted system as above but with additional 4.5 mM cCMP by monitoring the absorbance increase at 296 nm due to hydrolysis of cCMP upon adding drRNase A. Protein aggregation was monitored by recording the light scattering at 488 nm at 25°C in buffer A. To trace disulfide formation between Prx4 proteins and RNase A, aliquots of 40 µl reaction were blocked with 10 µl of 100 mM N-ethylmaleimide (NEM) at different time points, and analyzed in non-reducing SDS-12% PAGE followed by Coomassie blue staining and further Western blot using rabbit anti-Prx4 serum (Animal Facility, Institute of Genetics and Developmental Biology, Chinese Academy of Sciences, Beijing, China) and anti-RNase A antibody (Abcam), respectively.

## Results

### Prx4 can directly react with folding substrate in the presence of H_2_O_2_

**Figure 1:**
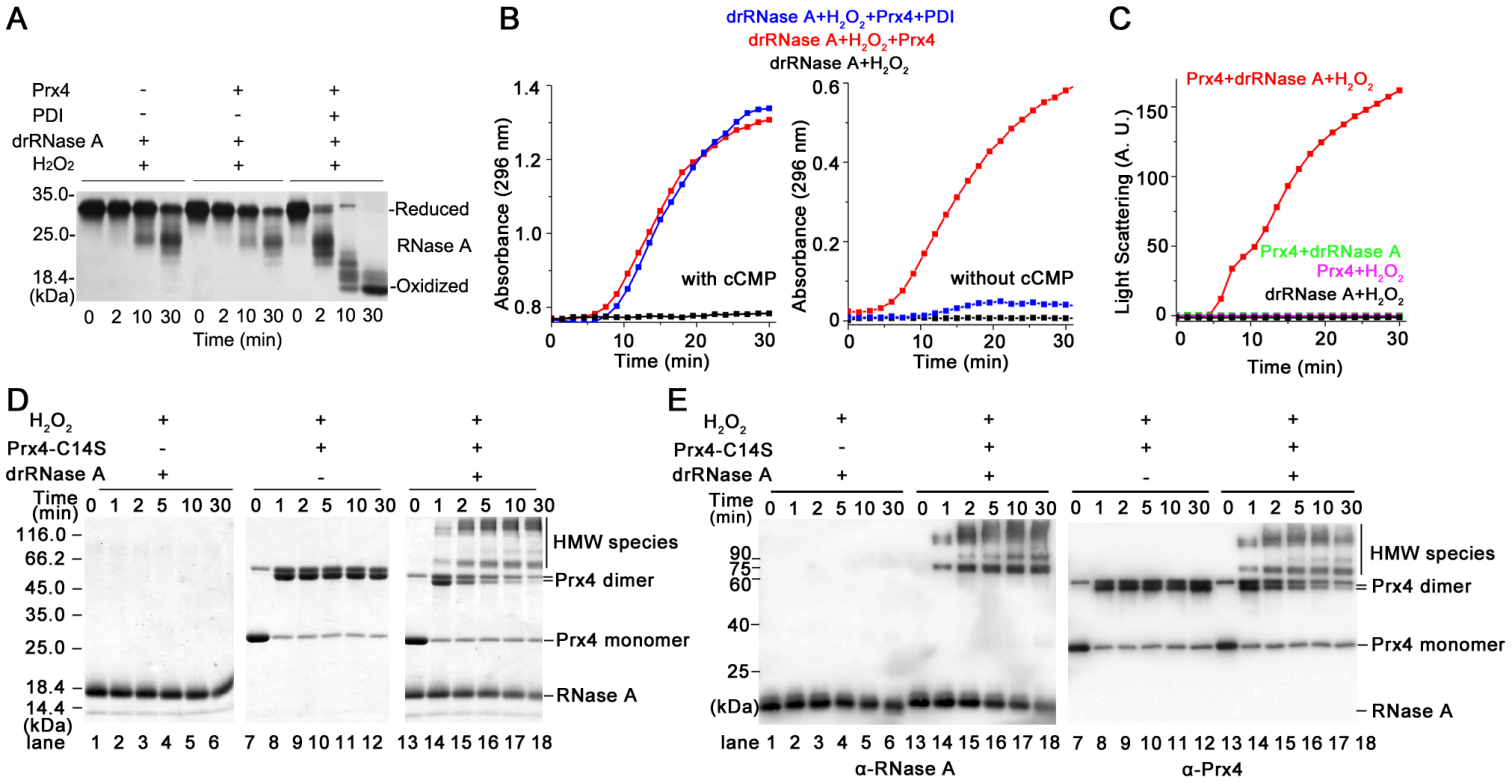
Direct reaction of Prx4 with substrate RNase A in the presence of H_2_O_2_. (**A**) Oxidative refolding of 8 µM drRNase A was carried out in buffer B containing 50 µM H_2_O_2_ in the presence or absence of 2.5 µM PDI and/or 2.5 µM Prx4 as indicated. Aliquots of reaction were quenched with 4-acetamido-4′-maleimidylstilbene-2,2′-disulfonic acid at indicated time points, and then analyzed by non-reducing SDS-15% PAGE followed by silver staining. (**B**) Reactivation of drRNase A was determined in the same system as in (A) with (left panel) or without (right panel) additional 4.5 mM cCMP by monitoring the absorbance increase at 296 nm due to cCMP hydrolysis. (**C**) Protein aggregation was monitored by recording the light scattering at 488 nm for the reactions of 2.5 µM Prx4, 50 µM H_2_O_2_ and/or 8 µM drRNase A in buffer A as indicated, respectively. A.U., arbitrary units. (**D**) Aliquots from the reaction of 2.5 µM Prx4-C14S and 8 µM drRNase A in the presence of 50 µM H_2_O_2_ at 25°C were removed and analyzed by non-reducing SDS-12% PAGE after alkylation with 20 mM NEM at the indicated time points. (**E**) The samples with the same numbering lane as in (D) were further Western blotted using anti-RNase A antibody and rabbit anti-Prx4 serum, respectively.

In our reconstituted refolding system composed of Prx4, H_2_O_2_ and PDI, drRNase A was almost fully oxidized (Figure 1A) and reactivated (Figure 1B). In a control experiment without PDI, a nearly identical absorbance increase at 296 nm as that with PDI was unexpectedly observed (Figure 1B, left), while no fully oxidized but only partially oxidized RNase A was detected (Figure 1A). This absorbance increase occurred even in the absence of cCMP, and disappeared only if PDI was present (Figure 1B, right). This intriguing absorbance increase was speculated as a result of aggregation, which was then confirmed by light scattering determination (Figure 1C). It was further found that the aggregation occurred only in the presence of Prx4, drRNase A and H_2_O_2_ altogether (Figure 1C).

Next we analyzed the reaction occurred during aggregation by non-reducing SDS-PAGE. The recombinant Prx4 itself was found to exist as monomers, disulfide-bonded dimers and also multimers on non-reducing SDS-PAGE [14], which would complicate the analysis. Since the disulfides via Cys^14^ between two dimers are unnecessary for peroxidase activity of Prx4 [8], we used the Prx4-C14S mutant, which exists only in monomeric and dimeric forms on non-reducing SDS-PAGE, for the disulfide status analysis in the following study. As shown in Figure 1D (lanes 13–18), high molecular weight (HMW) bands above Prx4-C14S dimer were formed and significantly increased during the reaction accompanied by attenuation of the Prx4-C14S monomer and dimer bands, meanwhile RNase A also slightly decreased (also see Figure 1E). As drRNase A (lanes 1–6, Figure 1D and 1E) or Prx4-C14S (lanes 7–12, Figure 1D and 1E) alone did not form the HMW species, the HMW species formed in the reaction (lanes 13–18, Figure 1D) must be disulfide cross-linked complexes between Prx4-C14S and RNase A, which were indeed recognized by both anti-Prx4 and anti-RNase A antibodies (lanes 13–18, Figure 1E).

**Figure 2:**
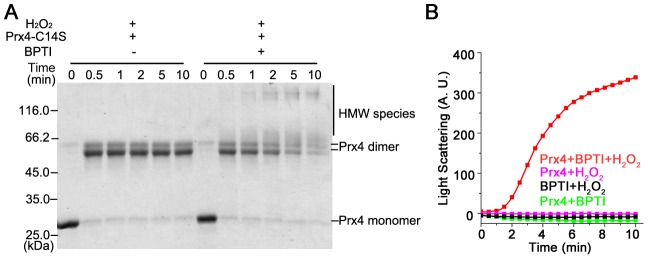
Direct reaction of Prx4 with substrate BPTI in the presence of H_2_O_2_. The reaction of 2.5 µM Prx4-C14S, 3 µM denatured and reduced BPTI and 50 µM H_2_O_2_ at 25°C in buffer A was analyzed by non-reducing SDS-12% PAGE (**A**) after alkylation with 20 mM NEM at the indicated time points, and protein aggregation during the reaction was monitored by light scattering at 488 nm (**B**).

We also checked the reaction of Prx4-C14S with another classical folding substrate, BPTI. As shown in Figure 2A, Prx4-C14S bands decreased with the emergence of disulfide cross-linked HMW species, and the rapid aggregation was also observed (Figure 2B). Therefore, the H_2_O_2_-dependent reactivity with folding substrates is very likely an inherent feature of Prx4.

### CysP-SOH form of Prx4 is responsible for disulfide formation with RNase A

**Figure 3:**
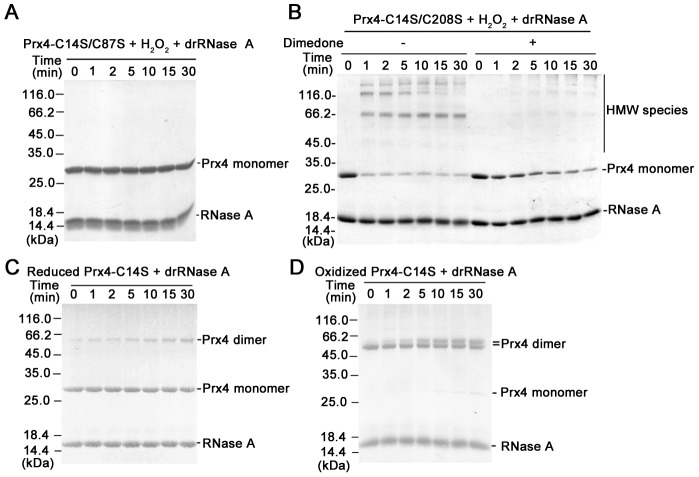
The CysP-SOH form of Prx4 is responsible for disulfide formation with RNase A. The reaction of 8 µM drRNase A with 2.5 µM Prx4-C14S/C87S and 50 µM H_2_O_2_ (**A**); or 2.5 µM Prx4-C14S/C208S and 50 µM H_2_O_2_ in the absence or presence of 15 mM dimedone (**B**); or 2.5 µM reduced Prx4-C14S (**C**) or oxidized Prx4-C14S (**D**) was carried out at 25°C in buffer A and analyzed by non-reducing SDS-12% PAGE after alkylation with 20 mM NEM at the indicated time points.

Prx4 contains 4 Cys residues. Cys^111^ is almost buried (PDB code 3TKP) and inaccessible, and Cys^14^ was found to be dispensable for the reaction with folding substrate (Figure 1D and Figure 2A). We then tested the involvement of Cys^87^ (CysP) and/or Cys^208^ (CysR) in the reaction. The Prx4-C14S/C208S mutant with CysP intact (Figure 3B and Figure S1), but not Prx4-C14S/C87S (Figure 3A), reacted to RNase A in the presence of H_2_O_2_ with rapid formation of disulfide cross-linked HMW species and caused protein aggregation (Figure S2), indicating that CysP but not CysR is essential for the reaction. Reduced Prx4-C14S (Figure 3C) and oxidized Prx4-C14S with the disulfide between CysP and CysR (Figure 3D) both failed to form disulfide cross-linked HMW species with RNase A. These results suggest that only the H_2_O_2_-dependent intermediate form of Prx4 (CysP-SOH) is responsible for the reaction with RNase A. This was further confirmed by the fact that in the presence of dimedone, a sulfenic acid-specific reagent [15], the formation of HMW species was significantly decreased (Figure 3B and Figure S1).

**Figure 4:**
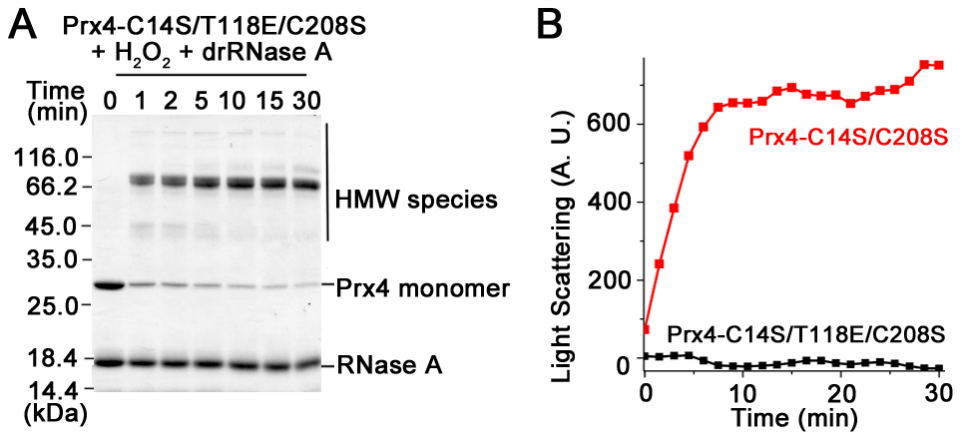
The decameric structure of Prx4 favors to form aggregation with RNase A. (**A**) The reaction of 2.5 µM Prx4-C14S/T118E/C208S, 8 µM drRNase A and 50 µM H_2_O_2_ at 25°C in buffer A was analyzed by non-reducing SDS-12% PAGE after alkylation with 20 mM NEM at the indicated time points. (**B**) Protein aggregation was monitored for the reaction of 8 µM drRNase A with 2.5 µM Prx4-C14S/C208S or Prx4-C14S/T118E/C208S in the presence of 50 µM H_2_O_2_ as indicated.

Prx4 is a 280-kDa decameric molecule with a CysP in each subunit, and the mutation of Thr^118^ to Glu results in the dissociation of decamer into dimer [14]. The Prx4-C14S/T118E/C208S mutant was detected to form mainly ∼70 kDa disulfide cross-linked complexes with RNase A (Figure 4A), which were recognized by both anti-Prx4 and anti-RNase A antibodies (Figure S3), and no aggregation was detected by light scattering (Figure 4B). The above data suggest the decameric structure of Prx4 might be in favor of promiscuous disulfide formation with folding substrate, leading to aggregation.

### PDI inhibits the reaction between Prx4 and substrates

**Figure 5:**
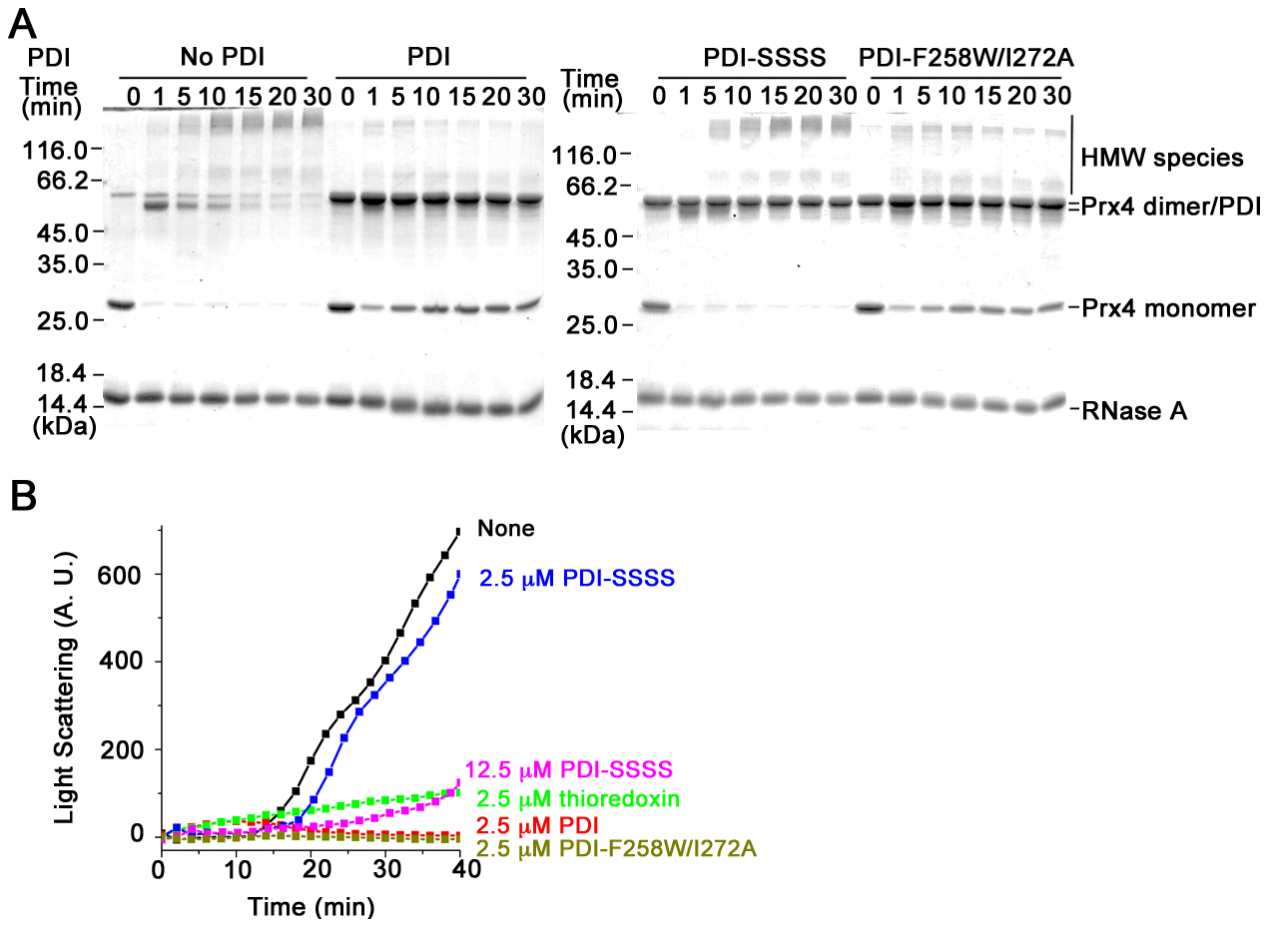
Inhibition of disulfide cross-linking and aggregation by PDI. (**A**) The reactions of 2.5 µM Prx4-C14S, 8 µM drRNase A and 50 µM H_2_O_2_ with or without 2.5 µM PDI proteins was analyzed by non-reducing SDS-12% PAGE after alkylation with 20 mM NEM at the indicated time points. (**B**) Protein aggregation in the reaction of 2.5 µM Prx4, 8 µM drRNase A and 50 µM H_2_O_2_ in the presence of PDI, reduced thioredoxin, PDI-SSSS, or PDI-F258W/I272A at indicated concentrations was monitored by recording light scattering.

The formation of disulfide cross-linked aggregate implies an inherent risk in the Prx4-mediated oxidative folding pathway. We found that the presence of PDI in the reconstituted system greatly stimulated the oxidation (Figure 1A) and reactivation of drRNase A (Figure 1B). Moreover, in the presence of PDI the disulfide cross-linked HMW species between Prx4-C14S and RNase A were gradually attenuated with an increase in monomeric Prx4 (Figure 5A), and the aggregation was almost suppressed in this process (Figure 5B). The above indicate that PDI plays a role in preventing the off-pathway aggregation by its reductase activity at least. In this respect, reduced *E. coli* Trx, an active reductase with the structure similar to the catalytic domain of PDI [16], is also capable of inhibiting the aggregation efficiently (Figure 5B). On the contrary, the PDI-SSSS mutant lacking all four cysteines in its two active sites, which loses reductase activity but retains chaperone activity [17], was ineffective to prevent the formation of HMW species (Figure 5A) and aggregation (Figure 5B). It is worthwhile to note that PDI-SSSS at a higher concentration showed considerable inhibitory effect (Figure 5B), suggesting that PDI inhibits the aggregation also by exerting its chaperone activity. The PDI-F258W/I272A mutant with impaired chaperone activity [18] but most of reductase activity (Figure S4) exhibited a similar effect as wild-type PDI in inhibiting the disulfide cross-linking reaction (Figure 5A) and attenuating the aggregation (Figure 5B). The above suggest that the reductase activity of PDI plays a major role with its chaperone activity as an auxiliary in inhibition of aggregation caused by the reaction between Prx4 and RNase A.

## Discussion

**Figure 6:**
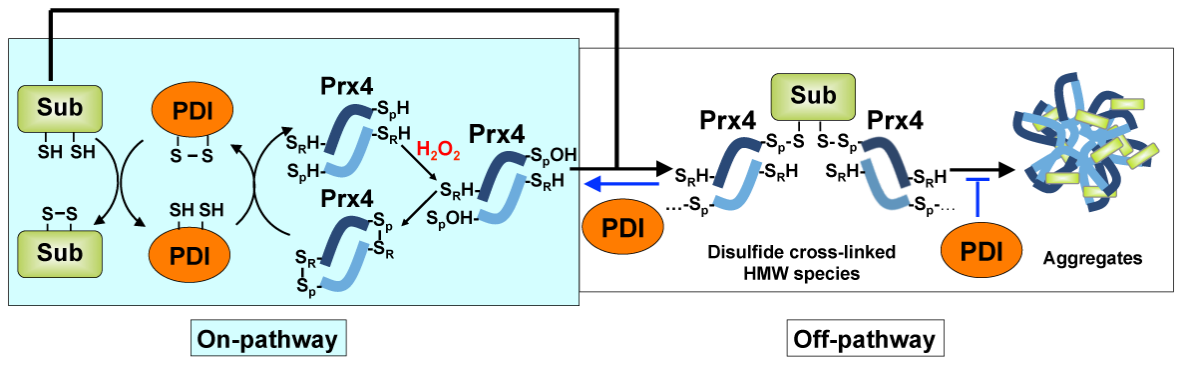
Schematic model of Prx4-mediated oxidative folding. Upon reaction with H_2_O_2_, CysP of reduced Prx4 is oxidized to -SOH state and then forms a disulfide bond with CysR from another subunit. The oxidized Prx4 transfers disulfides to folding substrate through PDI (on-pathway). The CysP-SOH form of Prx4 can also react with folding substrate, resulting in disulfide cross-linked HMW species and further aggregation (off-pathway). PDI plays additional quality control role to counterbalance the off-pathway reactions by exerting reductase and chaperone activities, ensuring efficient oxidative protein folding. Only the dimeric catalytic unit of Prx4 is presented for simplification. Sub, folding substrates.

The Ero1/PDI system has been established as the major pathway for oxidative protein folding in the eukaryotic ER [19], [20], producing H_2_O_2_ as a byproduct. ER-resident NADPH oxidases and mitochondrial respiration can also generate H_2_O_2_ [21]. Prx4 was recently found to be able to couple H_2_O_2_ removal with oxidative folding using PDI as an intermediary to transfer oxidizing equivalents to folding substrates [8], [11]. In this study, we report a unique reactivity of Prx4, that in the presence of H_2_O_2_ it can directly react with folding substrates via disulfide cross-linking, forming an off-pathway, which discloses a pitfall of the Prx4-mediated oxidative folding (Figure 6). In contrast, in the Ero1/PDI pathway Ero1 reacts only with PDI but not with folding substrates [3], [22]. Glutathione peroxidase 7 (GPx7), another peroxidase in the ER directly utilizing Ero1-generated H_2_O_2_ to promote oxidative protein folding [23], [24], neither forms disulfide cross-linking with folding substrates (data not shown).

Being a typical 2-Cys Prx, the active site of Prx4 alternates among reduced (-SH), sulfenic acid intermediate (-SOH) and disulfide states upon reaction with H_2_O_2_ (Figure 6). At high concentrations of H_2_O_2_, CysP-SOH may be overoxidized to form CysP-SO_2_H [5]. Here we discovered a new reaction feature of CysP, i.e. its -SOH form can directly react with thiols in folding substrates and form an intermolecular disulfide. This reaction is thus under the competition with the resolving of CysP-SOH by CysR in the on-pathway. In this respect, protein aggregation resulted from the reaction of Prx4-C14S/C208S with RNase A occurred much faster than that from wild-type Prx4 (Figure S2). This supported the previous report that Prx4 lacking its CysR is a poor oxidant of PDI and folding substrate RNase A [11] as it tends towards the off-pathway reaction.

Roles of PDI in Prx4-mediated oxidative folding are also highlighted here. PDI not only transfers disulfide between Prx4 and folding substrates in the on-pathway, but also helps avoid the pitfall by inhibiting the off-pathway reaction, by its reductase activity dominantly as well as its chaperone activity. Therefore PDI, being a foldase and a chaperone, ensures the high efficiency and fidelity of Prx4-mediated oxidative protein folding (Figure 6). Besides, we also found that PDI family members ERp46 and P5, which were recently reported to interact with Prx4 [25], also can inhibit the disulfide cross-linking between Prx4 and folding substrate (unpublished data).

In cells there should be kinetic competitions among the on-pathway oxidative protein folding, the off-pathway reaction with folding substrate and the quality control effect of PDI. In normal physiological conditions, abundant PDI in the ER [26] promotes the on-pathway oxidative protein folding and exerts its quality control role to efficiently prevent the possible Prx4-mediated off-pathway reaction. When vigorous protein synthesis occurred, the amount of reduced and unfolded nascent proteins and Ero1-generated H_2_O_2_ may exceed the capacity of the quality control system, which would lead the reaction towards off-pathway. Under aberrant conditions such as in Parkinson's or Alzheimer's diseases, loss of PDI function via S-nitrosylation and the consequent protein aggregation were found in the brain of patients [27]. Moreover, the disulfide cross-linked aggregates formed under oxidative stress have been also linked to the pathogenesis of neurodegenerative diseases [28], [29].

Interestingly, the newly established PspE/DsbC oxidative folding pathway in the periplasm of *E. coli* appears to share a similar reaction mechanism. PspE, a periplasmic rhodanese, could also react with other proteins via the sulfenic acid form of its single cysteine to generate disulfide cross-linked complexes, which are subsequently resolved by DsbC. Remarkably, the complex formation is significantly more pronounced in the absence of DsbC, the bacterial PDI [30].

## Supporting Information

Figure S1 **Western blot profile of the non-reducing SDS-PAGE in** **Figure 3B** **by using anti-Prx4 and anti-RNase A antibody respectively.**
(TIF)

Figure S2 **CysP of Prx4 is responsible for protein aggregation in its reaction with RNase A.** Protein aggregation for the reactions of 2.5 µM Prx4, Prx4-C14S/C87S or Prx4-C14S/C208S with 8 µM denatured and reduced RNase A and 50 µM H2O2 was monitored by recording the light scattering at 488 nm at 25°C. A.U., arbitrary units.
(TIF)

Figure S3 **Western blot profile of the non-reducing SDS-PAGE in** **Figure 4A** **by using anti-Prx4 and anti-RNase A antibody respectively.**
(TIF)

Figure S4 **Reductase activity of PDI and PDI-F258W/I272A.** The reductase activity of PDI was determined by monitoring insulin reduction. Insulin of 130 µM was added to 100 mM potassium phosphate buffer (pH 7.5) containing 2.5 mM EDTA and 100 µM DTT in presence of 2.5 µM PDI or PDI-F258W/I272A, and the absorbance increase at 650 nm due to light scattering of released and aggregated insulin B chain was recorded at 25°C. The reductase activity of PDI was calculated by the maximal slope of the curve relative to the lag time. The enzyme activity of wild-type PDI was taken as 100%. Data were expressed as mean ± S.D. (n = 3).
(TIF)
