# Supplementary figures and images for: A Novel Reaction of Peroxiredoxin 4 towards Substrates in Oxidative Protein Folding
Source: PLoS One. 2014 Aug 19;9(8):e105529. doi: 10.1371/journal.pone.0105529 (PMC4138195; doi:10.1371/journal.pone.0105529)

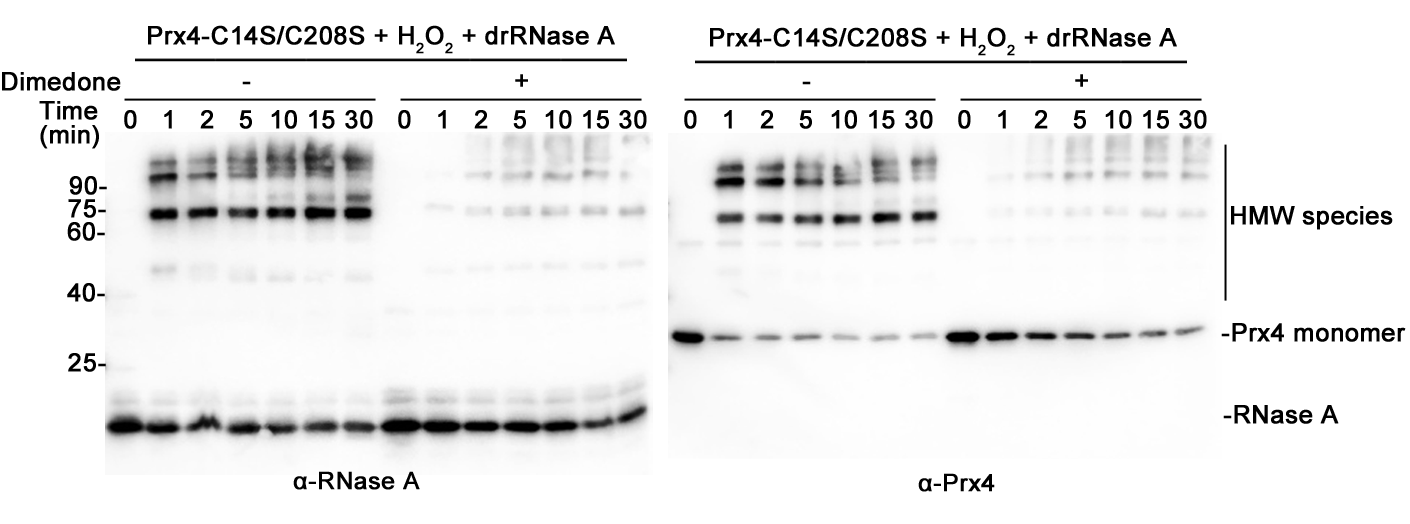

Supplement: Figure S1 — Western blot profile of the non-reducing SDS-PAGE in Figure 3B by using anti-Prx4 and anti-RNase A antibody respectively. (TIF) [file pone.0105529.s001.tif]

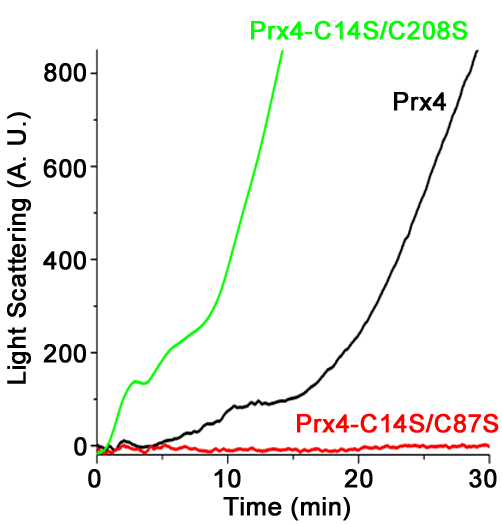

Supplement: Figure S2 — CysP of Prx4 is responsible for protein aggregation in its reaction with RNase A. Protein aggregation for the reactions of 2.5 µM Prx4, Prx4-C14S/C87S or Prx4-C14S/C208S with 8 µM denatured and reduced RNase A and 50 µM H2O2 was monitored by recording the light scattering at 488 nm at 25°C. A.U., arbitrary units. (TIF) [file pone.0105529.s002.tif]

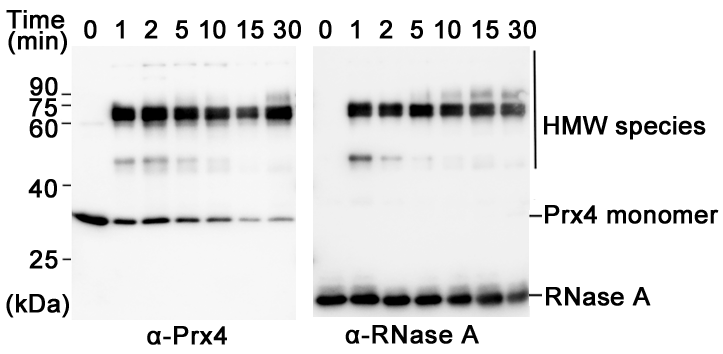

Supplement: Figure S3 — Western blot profile of the non-reducing SDS-PAGE in Figure 4A by using anti-Prx4 and anti-RNase A antibody respectively. (TIF) [file pone.0105529.s003.tif]

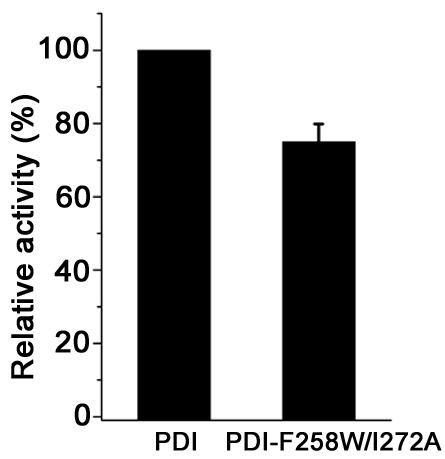

Supplement: Figure S4 — Reductase activity of PDI and PDI-F258W/I272A. The reductase activity of PDI was determined by monitoring insulin reduction. Insulin of 130 µM was added to 100 mM potassium phosphate buffer (pH 7.5) containing 2.5 mM EDTA and 100 µM DTT in presence of 2.5 µM PDI or PDI-F258W/I272A, and the absorbance increase at 650 nm due to light scattering of released and aggregated insulin B chain was recorded at 25°C. The reductase activity of PDI was calculated by the maximal slope of the curve relative to the lag time. The enzyme activity of wild-type PDI was taken as 100%. Data were expressed as mean ± S.D. (n = 3). (TIF) [file pone.0105529.s004.tif]
